# Mefenamic Acid-Upregulated Nrf2/SQSTM1 Protects Hepatocytes against Oxidative Stress-Induced Cell Damage

**DOI:** 10.3390/toxics11090735

**Published:** 2023-08-25

**Authors:** Wonseok Lee, Yewon Mun, Kang-Yo Lee, Jung-Min Park, Tong-Shin Chang, You-Jin Choi, Byung-Hoon Lee

**Affiliations:** College of Pharmacy and Research Institute of Pharmaceutical Sciences, Seoul National University, Seoul 08826, Republic of Korea; wonseokpharm@gmail.com (W.L.); munyewon98@snu.ac.kr (Y.M.); fuelforlife@naver.com (K.-Y.L.); parkjmking@hanmail.net (J.-M.P.); changts@snu.ac.kr (T.-S.C.)

**Keywords:** mefenamic acid, SQSTM1, Nrf2 pathway, oxidative stress, cytoprotective effect

## Abstract

Mefenamic acid (MFA) is a commonly prescribed non-steroidal anti-inflammatory drug (NSAID) with anti-inflammatory and analgesic properties. MFA is known to have potent antioxidant properties and a neuroprotective effect against oxidative stress. However, its impact on the liver is unclear. This study aimed to elucidate the antioxidative effects of MFA and their underlying mechanisms. We observed that MFA treatment upregulated the nuclear factor erythroid 2-related factor 2 (Nrf2) pathway. Treatment with various anthranilic acid derivative-class NSAIDs, including MFA, increased the expression of sequestosome 1 (SQSTM1) in HepG2 cells. MFA disrupted the interaction between Kelch-like ECH-associated protein 1 (Keap1) and Nrf2, activating the Nrf2 signaling pathway. SQTM1 knockdown experiments revealed that the effect of MFA on the Nrf2 pathway was masked in the absence of SQSTM1. To assess the cytoprotective effect of MFA, we employed tert-Butyl hydroperoxide (tBHP) as a ROS inducer. Notably, MFA exhibited a protective effect against tBHP-induced cytotoxicity in HepG2 cells. This cytoprotective effect was abolished when SQSTM1 was knocked down, suggesting the involvement of SQSTM1 in mediating the protective effect of MFA against tBHP-induced toxicity. In conclusion, this study demonstrated that MFA exhibits cytoprotective effects by upregulating SQSTM1 and activating the Nrf2 pathway. These findings improve our understanding of the pharmacological actions of MFA and highlight its potential as a therapeutic agent for oxidative stress-related conditions.

## 1. Introduction

Oxidative stress results from a disparity between the generation of reactive oxygen species (ROS) and the protective capacity of the antioxidant defense system. ROS is a toxic byproduct of energetic metabolism that can cause lipid peroxidation, DNA damage, and mitochondrial dysfunction if not adequately eliminated [1]. For example, 4-tert-butylphenol has been shown to induce apoptosis and necroptosis in grass carp hepatocytes, as evidenced by elevated cell death rates, disrupted mitochondria function, and increased ROS; these effects were mitigated by ROS scavenger pretreatment, implicating the ROS-PARP1 pathway [2]. Cadmium has been found to trigger heart damage through oxidative stress-mediated disruption of mitochondrial division/fusion, resulting in inflammation and autophagy [3]. Transcriptome analysis of tributyltin-exposed *Liza haematocheila* livers unveiled disrupted ATPase activities which resulted in oxidative stress, along with compromised antioxidant enzyme function, ultimately leading to liver apoptosis [4]. Consequently, regulating intracellular ROS level is crucial, as excessive ROS production has been implicated in various diseases [5].

Nuclear factor erythroid 2-related factor 2 (Nrf2) is a basic-region leucine zipper transcription factor that induces the expression of antioxidant genes, thereby protecting cells from oxidative stress [6]. Antioxidant and detoxification enzymes induced by Nrf2 include heme oxygenase 1 (HMOX1), NAD (P)H: quinone oxidoreductase 1 (NQO1), superoxide dismutase 1 (SOD1), and glutamate-cysteine ligase modifier subunit (GCLM). Nrf2 plays a pivotal role in protecting normal cells against oxidative stress. An elevated level of ROS creates deleterious conditions for normal cells, resulting in cellular damage. As a result, the activation of the Nrf2 pathway has garnered significant attention as a target for mitigating cellular injuries associated with oxidative stress, such as traumatic brain injury and preeclampsia [7,8]. However, it is noteworthy that hyperactivation of Nrf2 fosters an environment that is conducive to the growth and development of cancer cells [9,10,11]. This occurs through the inhibition of apoptosis, thereby facilitating cell proliferation and fortifying chemoresistance. Consequently, the Nrf2 pathway has emerged as a promising chemotherapeutic target for multiple cancer types, with the goal of sensitizing cancer cells to chemotherapy and potentially amplifying its clinical efficacy. While Nrf2 is expressed ubiquitously, its abundance is highest in metabolically active organs, particularly the liver [12]. Elevated ROS levels in the liver can cause hepatocellular damage and are associated with chronic liver disease [13]. Therefore, therapeutics targeting Nrf2 have shown promise in treating conditions such as non-alcoholic fatty liver disease and alcoholic liver disease [14,15].

The Kelch-like ECH-associated protein 1 (Keap1)-Nrf2 pathway serves as the primary regulator of cytoprotective responses against both endogenous and exogenous stresses induced by ROS [16]. Under normal conditions, Nrf2 is bound to Keap1 and sequestered in the cytoplasm. Keap1 also acts as an adaptor for Cullin 3-based E3 ligase, leading to the degradation of Nrf2 via polyubiquitination and subsequent proteasomal degradation [17]. During oxidative stress, Keap1 undergoes a conformational change, causing the release of Nrf2 from Keap1 and its translocation to the nucleus [18]. Additionally, sequestosome 1 (SQSTM1, also known as p62) positively regulates the Nrf2 pathway through the non-canonical pathway. In HEK293T cells, phosphorylated SQSTM1 at Ser349 has a higher binding affinity for Keap1 than for Nrf2, allowing it to compete with Keap1 for Nrf2 interaction, leading to Nrf2 dissociation from Keap1 [19]. Notably, the SQSTM1 promoter contains an antioxidant response element (ARE) which is responsible for its increased expression via Nrf2, thereby establishing a positive feedback loop in the SQSTM1-Keap1-Nrf2 pathway in HeLa cells [20]. Overall, SQSTM1 accumulation triggers Nrf2 nuclear translocation, resulting in further upregulation of SQSTM1 expression at the transcriptional level.

Non-steroidal anti-inflammatory drugs (NSAIDs), including the subclass of fenamates derived from anthranilic acid, are widely used to alleviate pain and inflammation [21]. Mefenamic acid (MFA) is one such NSAID. It has potent antioxidant properties and neuroprotective effects against oxidative stress [22]. MFA has also shown neuroprotective effects in Alzheimer’s disease animal models [23]. However, we have previously reported that MFA stimulates ROS generation in hepatocytes [24]. This observation has led us to hypothesize that the ROS generated by MFA might trigger the activation of the Nrf2 pathway, thereby potentially mediating the cytoprotective effect of MFA in hepatocytes. In this study, we aimed to elucidate the underlying molecular mechanisms responsible for the hepatoprotective effects of MFA against exogenous oxidative stress, as well as to report the sequence of events beginning with the initiation of the Nrf2 pathway, triggered by MFA-induced ROS generation, followed by the upregulation of SQSTM1 via the Nrf2 pathway. Treatment with N-acetyl-L-cysteine (NAC), an antioxidant, provides evidence of ROS-mediated SQSTM1 accumulation in MFA-treated hepatocytes. Here, we elucidate the existence of a positive feedback loop through the accumulation of SQSTM1 and provide in vitro evidence of cytoprotective effects of MFA in hepatocytes through Nrf2 activation. To mimic oxidative stress conditions, we employed tert-Butyl hydroperoxide (tBHP), a short-chain analogue of lipid peroxide, and palmitate. These agents are well-established for their ability to induce intracellular ROS accumulation in HepG2 cells, leading to cell death. Our findings provide valuable insight into the hepatoprotective effects of MFA against exogenous oxidative insult.

## 2. Materials and Methods

### 2.1. Cell Culture and Chemicals

Human hepatoma HepG2 cells were purchased from the American Type Culture Collection (ATCC, Manassas, VA, USA). HepG2 cells were grown in Dulbecco’s modified Eagle medium (Hyclone Laboratories, Logan, UT, USA, SH30021.01), supplemented with 10% heat-inactivated fetal bovine serum (Gibco, Carlsbad, CA, USA, 16000044) and 1% antibiotic-antimycotics (Gibco, Carlsbad, CA, USA, 15250062). Mefenamic acid (MFA; M1782) was purchased from Tokyo Chemical Industry (Tokyo, Japan), while 3-(4,5-dimethylthiazol-2-yl)-2,5-diphenyltetrazolium bromide (MTT; M6494), 5- (and-6)-chloromethyl-2′,7′-dichlorodihydrofluorescein diacetate (CM-H_2_DCFDA; C6827), and tetramethylrhodamine methyl ester (TMRM; T668) were purchased from Thermo Fisher Scientific (Waltham, MA, USA). tBHP (B2633), NAC (A9165), and palmitate (P5585) were purchased from Sigma Aldrich (St. Louis, MO, USA).

### 2.2. Chemical Treatment

HepG2 cells were treated with 5 or 10 mM NAC for 1 h, followed by MFA treatment at 200 µM for 24 h. To mimic oxidative stress conditions, cells were pre-treated with MFA at 200 µM for 1 h before a 24 h treatment with tBHP at 1 mM. All treatments were performed using the complete medium.

### 2.3. Western Blot and Immunoprecipitation

Cell pellets were lysed on ice for 90 min in buffer containing 50 mM HEPES, 150 mM NaCl, 5 mM EGTA, 50 mM β-glycerophosphate, 1% Triton-X 100, and protease inhibitor cocktail (Roche, Basel, Switzerland). After centrifugation, the supernatant was collected, electrophoresed on 6–8% sodium dodecyl sulfate-polyacrylamide gel, and transferred onto a polyvinylidene fluoride membrane (Millipore, Burlington, MA, USA). Transferred proteins were blocked in 5% skim milk, probed with primary antibodies at 4 °C overnight, and then incubated with HRP-conjugated secondary antibodies (Cell Signaling Technology, Danvers, MA, USA). Primary antibodies are shown in Table 1. Western blots were developed using Amersham ECL Prime Detection Reagent (GE Healthcare Life Sciences, Marlborough, IL, USA, RPN2232). Quantitative densitometry analysis was performed using ImageJ software 1.53t.

For immunoprecipitation, total cell lysates were conjugated with anti-Keap1 at 4 °C overnight. These conjugates were captured by protein G agarose beads (Millipore, Burlington, MA, USA, 16-266). The immunoprecipitated bead was washed with lysis buffer three times and boiled with SDS sample buffer. After centrifugation, the lysates were subjected to western blotting.

### 2.4. RNA Preparation and qRT-PCR Analysis

RNA was prepared from cells using an Easy-Blue Total RNA Extraction Kit (Intron Biotechnology, Seongnam, Gyeonggi, Republic of Korea, 17061). cDNA was synthesized using a QuantiTect Reverse Transcription Kit (Qiagen, Hilden, Germany, 205313). The resulting cDNA was amplified by qRT-PCR using iTaq Universal SYBR Green Supermix (Bio-Rad, Hercules, CA, USA, 1725121). The primers were produced by Cosmogenetech (Seoul, Republic of Korea) and the primer sequences are shown in Table 2.

### 2.5. Transient Transfections and Luciferase Reporter Gene Assay

HepG2 cells were transfected with small interfering RNA (siRNA) duplexes targeting human SQSTM1 (Bioneer, Seoul, Republic of Korea, 8878) or control siRNA using Lipofectamine RNAiMAX (Invitrogen, Carlsbad, CA, USA, 13778075). Specifically, HepG2 cells were transfected with 20 nM siSQSTM1 for 48 h, followed by incubation with 200 μM MFA for 24 h. For the luciferase reporter gene assay, cells were transfected with NQO1-ARE-luc (gifted from Dr. Sung Hwan Ki, Chosun University, Gwangju, Republic of Korea) using Lipofectamine 2000 (Invitrogen, Carlsbad, CA, USA, 11668019) according to the manufacturer’s protocols. pCMV-RL plasmids were co-transfected as an internal control. Luciferase activity was measured using a dual-luciferase reporter assay system (Promega, Madison, WI, USA, E1910). The luciferase activity was normalized with the renilla activity.

### 2.6. Nuclear Fractionation

For cytosolic extraction, buffer (20 mM HEPES (pH 7.0), 10 mM KCl, 1.5 mM MgCl_2_, 1 mM EDTA, 1 mM DTT, 0.1 mM PMSF, and 250 mM Sucrose) was added to cell pellets. After 30 min of incubation on ice, lysates were passed through 21G needles 25 times with a 1 mL syringe and centrifuged. The supernatant was collected as a cytosolic extract. The pellet was washed with buffet and then lysed with cell lysis buffer. After centrifugation, the supernatant was used as a nuclear fraction.

### 2.7. Measurement of Cell Viability, Intracellular ROS, and Mitochondrial Membrane Potential

Cells were cultured overnight and then treated with MFA or tBHP for 24 h. The next day, MTT was added to each well. After 2 h incubation, the formazan crystals were dissolved in dimethyl sulfoxide. The absorbance was examined at a wavelength of 570 nm using a microplate reader. To measure the cellular ROS levels, cells were incubated with CM-H_2_DCFDA fluorescent dye for 30 min. The fluorescence was measured using the Cytation 3 cell imaging multi-mode reader (Biotek, Winooski, VT, USA) at excitation and emission wavelengths of 480 nm and 520 nm, respectively. To assess mitochondrial membrane potential, cells were stained with TMRM. The fluorescence was measured at excitation and emission wavelengths of 548 nm and 574 nm, respectively.

### 2.8. Statistical Analysis

A statistical analysis of the data was performed using GraphPad Prism 9 (GraphPad Software, San Diego, CA, USA). Experimental data are presented as mean ± SD. Data were subjected to a Student’s *t*-test or one-way ANOVA analysis followed by a Tukey’s multiple comparison test. *p* value < 0.05 was considered statistically significant.

## 3. Results

### 3.1. MFA Activated the Nrf2 Pathway in HepG2 Cells

**Figure 1:**
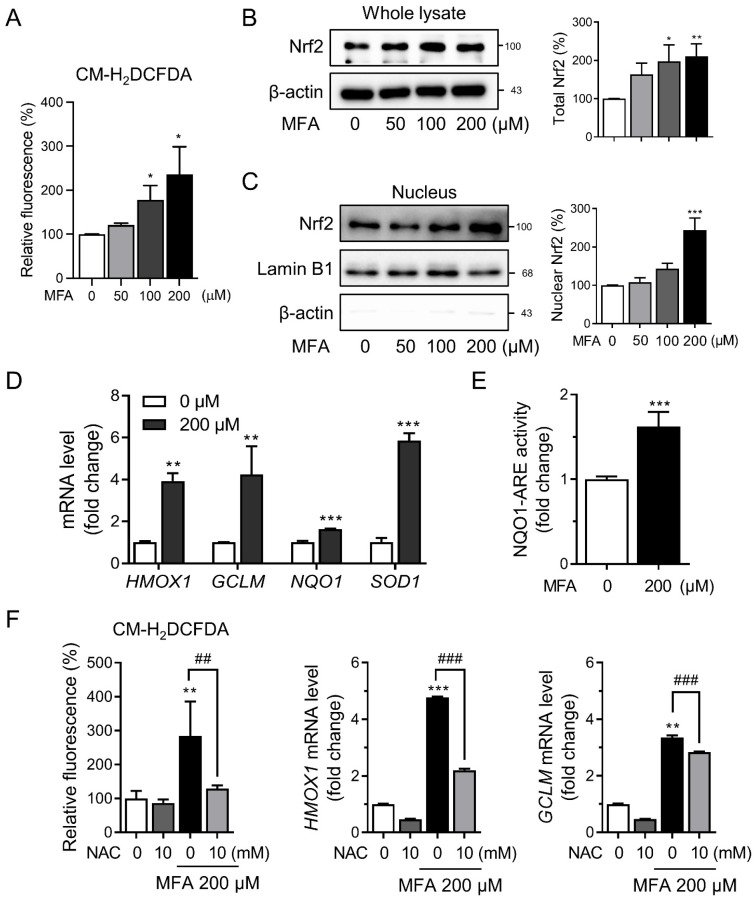
MFA activated the Nrf2 pathway. (**A**) HepG2 cells were treated with MFA for 1 h. Cellular ROS level was measured by CM-H_2_DCFDA. (**B**) HepG2 cells were treated with MFA for 24 h, and the level of Nrf2 was determined by western blot analysis. (**C**) The expression level of nucleus Nrf2 was measured by western blot analysis. Lamin B1 was used as a loading control for the nuclear protein fractions. (**D**) mRNA was extracted from HepG2 cells treated with MFA for 24 h, and mRNA levels of *HMOX1*, *GCLM*, *NQO1*, and *SOD1* were analyzed by qRT-PCR. (**E**) HepG2 cells were co-transfected with the reporter plasmid NQO1-ARE-luc and renilla plasmid and then treated with MFA for 24 h. Luciferase activities were determined using a dual-luciferase reporter assay system. (**F**) HepG2 cells were pretreated with NAC for 1 h before MFA treatment for 24 h. The left panel shows that cellular ROS levels were measured by CM-H_2_DCFDA. The mRNA levels of *HMOX1* and *GCLM* were assessed by qRT-PCR. Data are presented as mean ± SD of at least three independent experiments, as analyzed by one-way ANOVA followed by Tukey’s test. * *p* < 0.05, ** *p* < 0.01, and *** *p* < 0.001, relative to the control group. ## *p* < 0.01, and ### *p* < 0.001 relative to the MFA-treated group.

Previous studies have shown that various NSAIDs can induce the production of ROS [25,26]; for example, our previous study showed that diclofenac promoted mitochondrial ROS generation in hepatocytes [24]. In this study, we explored the effect of MFA on intracellular ROS accumulation in HepG2 cells using CM-H_2_DCFDA, a fluorescent dye that is sensitive to oxidation. We found that MFA significantly induced ROS production in HepG2 cells (Figure 1A). Additionally, MFA treatment resulted in a marked increase in the expression of Nrf2 (Figure 1B) and prompted the translocation of Nrf2 into the nucleus (Figure 1C). Moreover, MFA upregulated the mRNA expression of *HMOX1*, *GCLM*, *NQO1*, and *SOD1*, which are downstream targets of Nrf2 (Figure 1D). To confirm Nrf2-mediated induction, we used a reporter gene construct, NQO1-ARE-luc, and observed a significant increase in NQO1-luciferase activity upon MFA treatment (Figure 1E). Co-treatment with MFA and NAC, a well-known ROS scavenger, attenuated the induction of Nrf2 target genes by MFA (Figure 1F), indicating the involvement of intracellular ROS production in MFA-induced Nrf2 activation.

### 3.2. MFA Increased SQSTM1 Expression in HepG2 Cells

**Figure 2:**
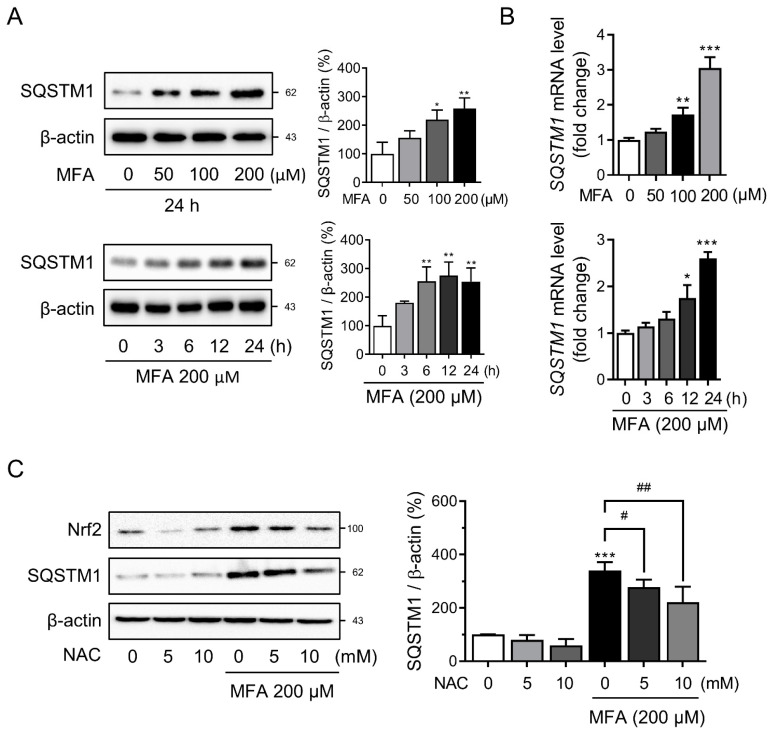
MFA increased the expression of SQSTM1 in HepG2 cells. (**A**) HepG2 cells were treated with MFA as indicated and subjected to western blot analysis with an antibody against SQSTM1; the densitometric quantification of SQSTM1 is shown. (**B**) HepG2 cells were incubated with MFA as indicated, and mRNA was isolated and subjected to qRT-PCR analysis to measure *SQSTM1* mRNA expression. (**C**) HepG2 cells were pretreated with NAC for 1 h before MFA treatment for 24 h. The protein levels of Nrf2 and SQSTM1 were measured by western blot analysis; the densitometric quantification of SQSTM1 is shown in the right panel. Data are presented as mean ± SD of at least three independent experiments, as analyzed by one-way ANOVA followed by Tukey’s test. * *p* < 0.05, ** *p* < 0.01, and *** *p* < 0.001, relative to the control group. # *p* < 0.05, and ## *p* < 0.01, relative to the MFA-treated group.

We investigated the molecular mechanisms underlying MFA-induced Nrf2 activation. The promoter region of SQSTM1 contains an ARE, and Nrf2 can induce SQSTM1 transcription in response to oxidative stress, thereby creating a positive feedback loop [16,20]. MFA treatment led to a dose- and time-dependent increase in SQSTM1 protein levels (Figure 2A). Furthermore, MFA significantly upregulated the mRNA expression of *SQSTM1* (Figure 2B), indicating transcriptional regulation of SQSTM1. Other NSAIDs from the anthranilic acid family, such as flufenamic acid, meclofenamic acid, and tolfenamic acid, also showed similar results (Supplementary Figure S1). Co-treatment with NAC attenuated MFA-induced SQSTM1 induction, suggesting that intracellular ROS production was a significant factor in MFA-mediated SQSTM1 induction (Figure 2C). It has been reported that NSAIDs, such as celecoxib, can induce an elevation in the expression of LC3-II and p62. This effect is achieved through the inhibition of lysosomal function, resulting in the suppression of autophagy [27]. Treatment with MFA did not affect the protein levels of autophagic markers LC3B-II and NBR1 (Supplementary Figure S2), indicating that MFA-induced SQSTM1 expression was independent of autophagic activity.

### 3.3. SQSTM1 Was Required for MFA-Induced Nrf2 Activation

**Figure 3:**
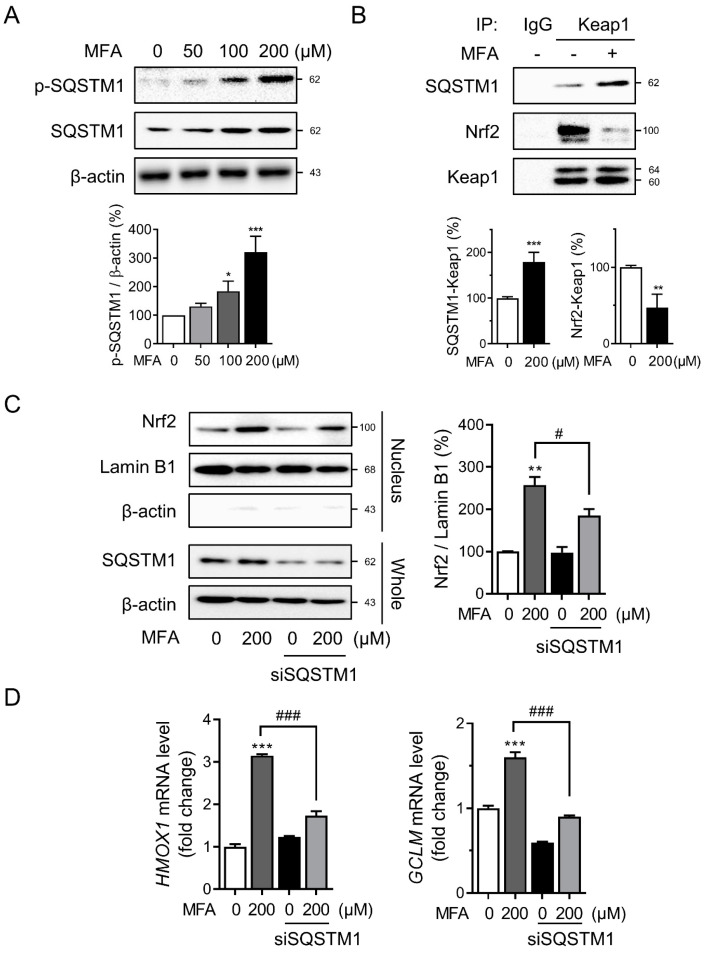
SQSTM1 was required for MFA−induced Nrf2 activation. (**A**) Western blot analyses of p−SQSTM1 and SQSTM1 in HepG2 cells treated with MFA for 24 h are shown. (**B**) HepG2 cells were treated with MFA (200 μM) for 24 h. Immunoprecipitations were performed using IgG or anti-Keap1 antibodies and then analyzed by western blot analysis using the indicated antibodies. (**C**,**D**) HepG2 cells were transfected with control or SQSTM1 siRNA and were treated with MFA for 24 h. (**C**) Whole cell lysate or nuclear protein fractions were subjected to western blot analysis. Lamin B1 was used as a loading control for the nuclear protein fractions. The right panel showed the quantification of the nucleus Nrf2 level. (**D**) The mRNA levels of *HMOX1* and *GCLM* were assessed by qRT−PCR. Data are presented as mean ± SD of at least three independent experiments, as analyzed by one-way ANOVA followed by Tukey’s test. * *p* < 0.05, ** *p* < 0.01, and *** *p* < 0.001, relative to the control group. # *p* < 0.05, and ### *p* < 0.001, relative to the MFA−treated group.

The interaction between p62 and Keap1 stabilizes Nrf2 by decreasing CRL3-Keap1 mediated ubiquitination, which allows for Nrf2-mediated activation of the antioxidant response pathway [19]. Phosphorylation of SQSTM1 enhances its interaction with Keap1, leading to the release of Nrf2 from Keap1 and the transcriptional upregulation of Nrf2 target genes [28]. Thus, we examined the phosphorylation of SQSTM1 in HepG2 cells treated with MFA and found that MFA increased both the expression and phosphorylation of SQSTM1 (Figure 3A). Co-immunoprecipitation revealed an increased interaction between SQSTM1 and Keap1, while the interaction between Nrf2 and Keap1 decreased following MFA treatment (Figure 3B). To confirm the involvement of SQSTM1 in MFA-induced Nrf2 activation, we used siRNA to knock down SQSTM1 in HepG2 cells. Knockdown of SQSTM1 inhibited the increased nuclear translocation of Nrf2 induced by MFA (Figure 3C), and it also downregulated the induction of Nrf2 target genes, including HMOX1 and GCLM (Figure 3D). These results indicate that SQSTM1 plays a role in MFA-induced Nrf2 activation by modulating the interaction between Keap1 and Nrf2.

### 3.4. MFA Alleviated tBHP-Induced Cytotoxicity via SQSTM1-Mediated Nrf2 Activation

**Figure 4:**
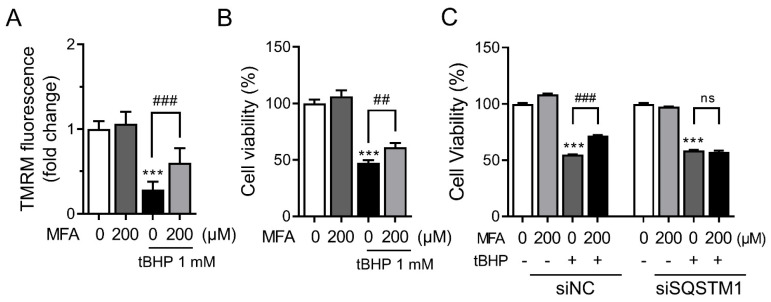
MFA alleviated tBHP−induced cytotoxicity via SQSTM1−mediated Nrf2 activation. (**A**) HepG2 cells were pretreated with MFA (200 µM) for 1 h and further incubated with tBHP (1 mM) for 24 h. Mitochondrial membrane potential was analyzed using TMRM dye. (**B**) HepG2 cells were pretreated with MFA (200 µM) for 1 h and further incubated with tBHP (1 mM) for 24 h. Cell viability was determined by MTT assay. (**C**) HepG2 cells were transfected with control or SQSTM1 siRNA. Cells were pretreated with MFA (200 µM) for 1 h and further incubated with tBHP (1 mM) for 24 h. Cell viability was determined by MTT assay. Data are presented as mean ± SD of at least three independent experiments, as analyzed by one-way ANOVA followed by Tukey’s test. *** *p* < 0.001, relative to the control group. ## *p* < 0.01 and ### *p* < 0.001, relative to the indicated group. ns: non-significant relative to the tBHP−treated group.

Finally, we sought to determine whether MFA could protect HepG2 cells from oxidative stress-induced cytotoxicity. Treatment with MFA for 24 h significantly protected the cells from tBHP-induced cytotoxicity (Figure 4A). Similarly, MFA treatment reversed the increased cell death induced by palmitate (Supplementary Figure S3), which causes lipotoxicity through oxidative stress. Disruption of mitochondrial membrane potential serves as an indicator of oxidative stress-induced cell death via a mitochondria-dependent pathway [29]. We measured the relative fluorescence of the potential-sensitive probe TMRM and found that mitochondrial membrane potential decreased in tBHP-treated HepG2 cells, which was attenuated by MFA (Figure 4B). Moreover, silencing SQSTM1 abolished the protective effect of MFA against tBHP-induced oxidative damage (Figure 4C). These findings indicated that MFA prevents cell death induced by tBHP, and the activation of Nrf2 through SQSTM1 is responsible for the cytoprotective effect of MFA.

## 4. Discussion

Hepatic cell death is primarily caused by ROS and oxidative stress, which disrupt liver redox homeostasis and contribute to the progression of liver damage [30]. In our study, we induced oxidative stress in HepG2 cells using tBHP and palmitate. tBHP generates free radical intermediates, leading to the peroxidation of membrane lipids [31]. Ultimately, tBHP causes mitochondrial dysfunction in hepatocytes [32,33]. Palmitate, a saturated long-chain fatty acid, induces lipotoxicity in hepatocytes [34]. In response to oxidative stress, hepatocytes activate the Nrf2 transcription factor to induce the expression of antioxidant genes [35]. Dysregulation of Nrf2 can exacerbate liver diseases, including fatty liver, hepatitis, and hepatocellular carcinoma [36,37,38]. Therefore, the activation of Nrf2 could be a potential therapeutic target for the treatment of liver disease. Our study demonstrated that MFA treatment protected hepatocytes from oxidative stress-mediated cell damage by activating Nrf2. MFA induces the expression of downstream antioxidant enzymes, including HO-1 and GCLM, and reduces cell death and mitochondrial dysfunction caused by tBHP and palmitate treatment. To the best of our knowledge, this is the first study to demonstrate that MFA can protect hepatocytes from tBHP-induced cell damage through Nrf2 activation.

NSAIDs, including the anthranilic acid derivative class (e.g., MFA), are commonly used for pain relief and inflammation management [39,40]. Despite their widespread use as analgesics and anti-inflammatory therapeutics, the exact molecular mechanism of action of these drugs remains not fully understood. Although earlier studies have indicated that MFA can stimulate ROS generation [24,41], other studies have presented evidence of the ability of MFA to alleviate oxidative stress and confer neuroprotection [22,23]. In our investigation, we have illustrated that MFA initiates the Nrf2 pathway by inducing ROS production, leading to the upregulation of SQSTM1 via the Nrf2 pathway. This process involves a positive feedback loop where the upregulated SQSTM1 by the Nrf2 pathway further enhances Nrf2 activation. Consequently, Nrf2 activation by MFA plays a crucial role in protecting hepatocytes against tBHP-induced cell damage, providing significant cytoprotective effects. These results offer valuable insights into the hepatoprotective potential of MFA against oxidative insult.

Previous studies have reported that NSAIDs can generate ROS in the liver. In our previous research, we demonstrated that diclofenac enhances mitochondrial ROS generation in hepatocytes [24]. Similarly, Ahmad et al. reported that naproxen induces ROS-mediated hepatic lipid peroxidation [42]. Our current study supports these findings by showing that MFA elevates intracellular ROS levels in HepG2 cells. However, the impact of drug-induced ROS on cell survival is still a subject of debate [43]. ROS can serve as both signaling molecules and potentially harmful agents that induce cell death. The effects of ROS on cells depend on their concentration. Excessive ROS generation can overwhelm cellular protective mechanisms and lead to cell death, while manageable levels of ROS can activate antioxidant pathways, including the Nrf2 pathway [44]. In the present study, MFA was shown to exert a cytoprotective effect against oxidative stress by increasing the expression of Nrf2 target genes. These results shed light on the mechanism by which MFA exhibits antioxidant activity.

The Keap1-Nrf2 regulatory pathway plays a central role in cellular defense against electrophilic and oxidative stress [45]. Nrf2 activation occurs when the interaction between Nrf2 and Keap1 is disrupted. Under normal conditions, Nrf2 is predominantly located in the cytosol, bound to Keap1, and constitutively degraded via the proteasome. During oxidative stress, conformational changes in the cysteine residues of Keap1 disrupt the Nrf2–Keap1 interaction and reduce Nrf2 degradation [18]. Similarly, the SQSTM1 protein acts as a positive regulator of the Nrf2 pathway [46]. Ichimura et al. demonstrated that the accumulation of phosphorylated SQSTM1 competitively binds to Keap1, resulting in the transcriptional activation of Nrf2 target genes [28]. Consistent with these findings, our data show that MFA induces the phosphorylation of SQSTM1, thereby promoting the dissociation of Nrf2 from Keap1.

SQSTM1 is a multifunctional, stress-inducible scaffold protein involved in signal transduction, cell survival, cell death, and oxidative stress response [47]. It serves as a cargo receptor for the selective autophagy of ubiquitinated target proteins, linking polyubiquitinated proteins with autophagy [48]. SQSTM1 has been identified as a key mediator in activating signaling pathways that regulate cell survival, including the Nuclear factor kappa B (NF-κB) and the mammalian target of rapamycin (mTOR) pathways [49,50]. However, in our study, although MFA was shown to increase the level of SQSTM1, autophagic activity remained unaltered, suggesting that mechanisms independent of autophagy contribute to the upregulation of SQSTM1. SQSTM1 also maintains the activation of Nrf2 through a positive feedback loop in the SQSTM1-Nrf2-Keap1 pathway [20]. Our research demonstrates that MFA increases the mRNA level of SQSTM1 while NAC reverses the expression of Nrf2 induced by MFA (Figure 2C). Moreover, the knockdown of SQSTM1 abolishes MFA-induced Nrf2 activation and nuclear translocation. These results imply that MFA-induced oxidative stress could lead to the elevation of SQSTM1 through the Nrf2 pathway, thereby establishing a positive feedback loop that subsequently enhances further Nrf2 activation.

## 5. Conclusions

**Figure 5:**
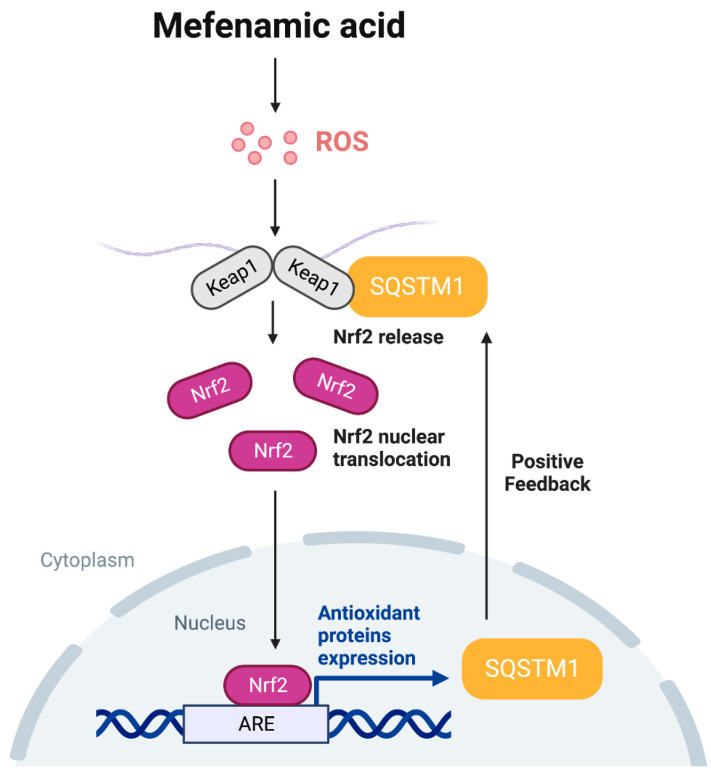
The underlying mechanisms of the protective effect of mefenamic acid against oxidative stress-induced cell damage through the Nrf2-SQSTM1 pathway.

In conclusion, our study has presented novel mechanisms of the cytoprotective effects of MFA against oxidative insult. MFA activates the Nrf2 pathway by inducing ROS. This, in turn, upregulates SQSTM1 through the Nrf2 pathway, leading to the accumulation of SQSTM1 that competes with Keap1. As a result, this process transcriptionally activates Nrf2 target genes, establishing a positive feedback loop that further enhances Nrf2 activation (Figure 5). Importantly, our research demonstrates that MFA effectively protects HepG2 cells against tBHP-induced oxidative injury by promoting Nrf2 activation. These findings collectively highlight the hepatoprotective effect of MFA and suggest its potential as a therapeutic target for liver diseases associated with oxidative stress.

## Figures and Tables

**Table 1:** List of primary antibodies.

Antibody	Dilution	Company	Catalog #
anti-SQSTM1	1:1000	Cell Signaling Technology	5114S
anti-Keap1	1:1000	Cell Signaling Technology	4678S
anti-p-SQSTM1	1:1000	MBL Life Science	PM074
anti-Nrf2	1:1000	Santa Cruz Biotechnology	sc-365949
anti-β-actin	1:5000	Santa Cruz Biotechnology	sc-47778
anti-Lamin B1	1:2000	Santa Cruz Biotechnology	Sc-374015

**Table 2:** List of primers for qRT-PCR.

Gene	Forward Primer (5′ to 3′)	Reverse Primer (5′ to 3′)
*SQSTM1*	GACCCACAGGGCTGAAGGAA	CCAGCCGCCTTCATCAGAGA
*HMOX1*	TCCGATGGGTCCTTACACTC	TAAGGAAGCCAGCCAAGAGA
*GCLM*	CATTTACAGCCTTACTGGGAGG	ATGCAGTCAAATCTGGTGGCA
*NQO1*	GAAGAGCACTGATCGTACTGGC	GGATACTGAAAGTTCGCAGGG
*SOD1*	GGTGGGCCAAAGGATGAAGAG	CCACAAGCCAAACGACTTCC
*ACTB*	CACCATTGGCAATGAGCGGTTC	AGGTCTTTGCGGATGTCCACGT

## Data Availability

The data presented in this study are available on request from the corresponding author.

## Supplementary Materials

The following supporting information can be downloaded at: https://www.mdpi.com/article/10.3390/toxics11090735/s1, Figure S1: Anthranilic acid derivative class NSAID drugs increase the level of SQSTM1 in HepG2 cells; Figure S2: The effects of MFA on SQSTM1 induction are independent of autophagic activity; Figure S3: MFA attenuates palmitate-mediated lipotoxicity in HepG2 cells.
