# Supplementary material for: Mefenamic Acid-Upregulated Nrf2/SQSTM1 Protects Hepatocytes against Oxidative Stress-Induced Cell Damage
Source: Toxics. 2023 Aug 25;11(9):735. doi: 10.3390/toxics11090735 (PMC10536671; doi:10.3390/toxics11090735)
Supplement: Supplementary file 1 [file toxics-11-00735-s001.zip › toxics-2555993-supplementary.pdf]

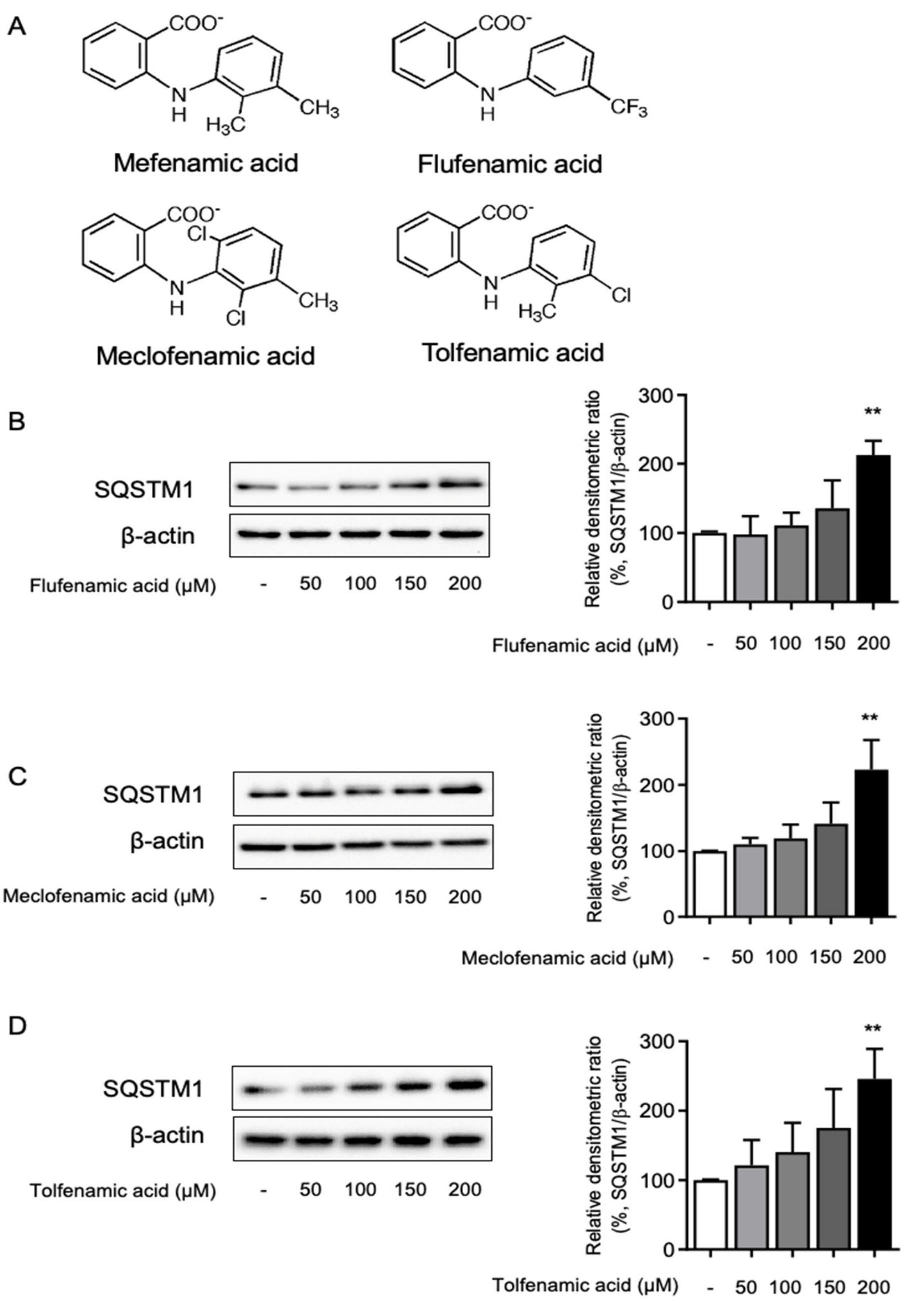

Figure S1. Anthranilic acid derivative class NSAID drugs increase the level of SQSTM1 in HepG2 cells. (A) Chemical structures of anthranilic acid derivative class NSAIDs tested in this study are shown. (B, C, D) HepG2 cells were incubated for 24 h with the indicated concentration of flufenamic acid, meclofenamic acid or tolfenamic acid. The protein levels of SQSTM1 were measured by western blot analysis. Representative western blot images and the relative quantification of proteins are shown. Data are presented as mean ± SD of at least 3 independent experiments, as analyzed by one-way ANOVA followed by Tuckey's test. ** $p < 0.01$, relative to the control group.

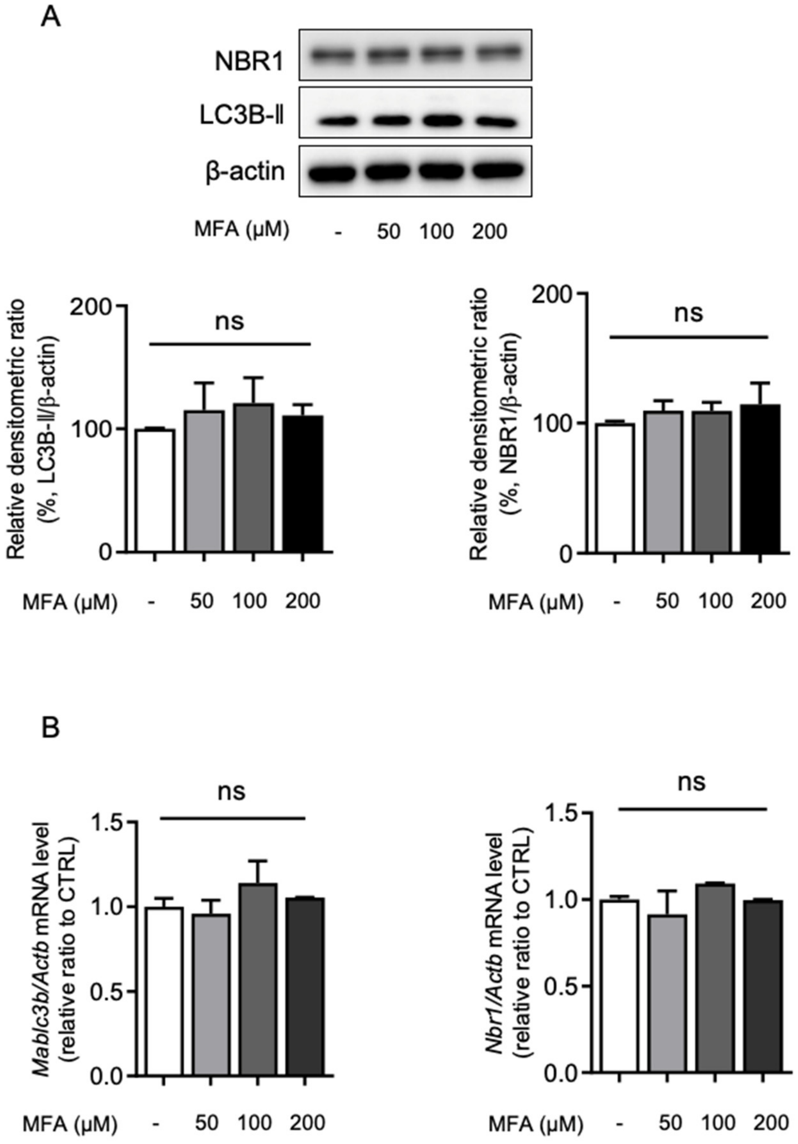

Figure S2. The effects of MFA on SQSTM1 induction are independent of autophagic activity. (A) HepG2 cells were treated with the indicated concentration of MFA (24 h) and subjected to western blot analysis with antibodies against LC3B-II and NBR1. Representative western blot images and the densitometric quantification of proteins are shown. (B) mRNA was extracted from HepG2 cells treated with MFA for 24 h, and mRNA levels of *Mablc3b* and *NBR1* were analyzed by qRT-PCR. Cell viability was determined by MTT assay. Data are presented as mean ± SD of at least 3 independent experiments, as analyzed by one-way ANOVA followed by Tuckey's test. ns; non-significant relative to the control group.

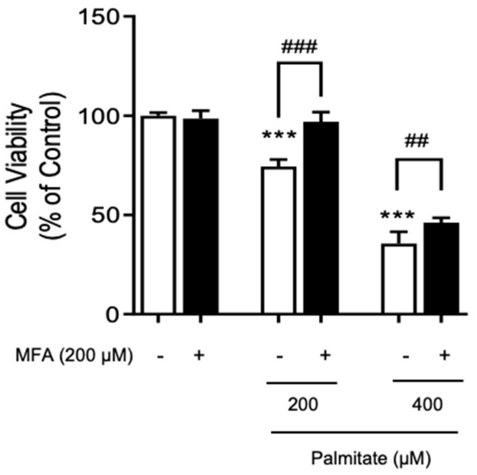

Figure S3. MFA attenuates palmitate-mediated lipotoxicity in HepG2 cells. HepG2 cells were pre-treated with MFA (200 μM) for 1 h, followed by palmitate (200, 400 μM) for 24 h. Cell viability was determined by MTT assay. Data are presented as mean ± SD of at least 3 independent experiments, as analyzed by one-way ANOVA followed by Tuckey's test *** $p < 0.001$, relative to the control group. ## $p < 0.01$, and ### $p < 0.001$, relative to the indicated group.
